# The Parkinson's disease-associated protein DJ-1 plays a positive nonmitochondrial role in endocytosis in *Dictyostelium* cells

**DOI:** 10.1242/dmm.028084

**Published:** 2017-10-01

**Authors:** Suwei Chen, Sarah J. Annesley, Rasha A. F. Jasim, Vanessa J. Musco, Oana Sanislav, Paul R. Fisher

**Affiliations:** 1 Department of Microbiology, Faculty of Science, Technology and Engineering, La Trobe University, VIC 3086, Australia; 2 School of Modern Agriculture and Biological Science and Technology, Ankang University, Shaanxi 725000, PRC; 3 Department of Laboratory and Clinical Sciences, College of Pharmacy, University of Babylon, PO Box 4, Hilla, Iraq

**Keywords:** *Dictyostelium*, DJ-1, Endocytosis, Macropinocytosis, Mitochondrial dysfunction, Parkinson's disease, Phagocytosis, Respiration

## Abstract

The loss of function of DJ-1 caused by mutations in *DJ1* causes a form of familial Parkinson's disease (PD). However, the role of DJ-1 in healthy and in PD cells is poorly understood. Even its subcellular localization in mammalian cells is uncertain, with both cytosolic and mitochondrial locations having been reported. We show here that DJ-1 is normally located in the cytoplasm in healthy *Dictyostelium discoideum* cells. With its unique life cycle, straightforward genotype-phenotype relationships, experimental accessibility and genetic tractability, *D.* *discoideum* offers an attractive model to investigate the roles of PD-associated genes. Furthermore, the study of mitochondrial biology, mitochondrial genome transcription and AMP-activated protein kinase-mediated cytopathologies in mitochondrial dysfunction have been well developed in this organism. Unlike mammalian systems, *Dictyostelium* mitochondrial dysfunction causes a reproducible and readily assayed array of aberrant phenotypes: defective phototaxis, impaired growth, normal rates of endocytosis and characteristic defects in multicellular morphogenesis. This makes it possible to study whether the underlying cytopathological mechanisms of familial PD involve mitochondrial dysfunction. DJ-1 has a single homologue in the *Dictyostelium* genome. By regulating the expression level of DJ-1 in *D. discoideum*, we show here that in unstressed cells, DJ-1 is required for normal rates of endocytic nutrient uptake (phagocytosis and, to a lesser extent, pinocytosis) and thus growth. Reduced expression of DJ-1 had no effect on phototaxis in the multicellular migratory ‘slug’ stage of the life cycle, but resulted in thickened stalks in the final fruiting bodies. This pattern of phenotypes is distinct from that observed in *Dictyostelium* to result from mitochondrial dyfunction. Direct measurement of mitochondrial respiratory function in intact cells revealed that DJ-1 knockdown stimulates whereas DJ-1 overexpression inhibits mitochondrial activity. Together, our results suggest positive roles for DJ-1 in endocytic pathways and loss-of-function cytopathologies that are not associated with impaired mitochondrial function.

## Abstract (precis)

**Editor's choice:** The *Dictyostelium* homologue of the Parkinson's disease-associated protein DJ-1 is located in the cytosol, and its loss causes cytopathological defects in endocytic and autophagic cell death pathways, but stimulates respiration by functionally normal mitochondrial respiratory complexes.

## INTRODUCTION

Attention was first drawn to the role of DJ-1 in Parkinson's disease (PD) with the report by Bonifati et al. (2003) of the first two DJ-1-related PD cases, involving a 14 kb deletion from a Dutch kindred and an L166P mutation from an Italian background. Human DJ-1 is a protein of 189 amino acids encoded by the ∼24 kb *DJ1* gene (also known as PARK7) at chromosome 1p36. Although the early-onset PD caused by deletions or mutations of DJ-1 appears to be rare, Choi et al. (2006) suggested that DJ-1 might also play a crucial role in sporadic late-onset PD, the majority of PD cases, because greater oxidative damage to DJ-1 and elevated DJ-1 protein levels were found in the postmortem brains of sporadic PD patients. Therefore, it is very important to understand how DJ-1 exerts its function both in healthy cells and in familial and idiopathic PD.

Various putative functions of DJ-1 in PD have been proposed, including roles as a redox-sensitive chaperone, antioxidant, transcriptional regulator, protease and protein deglycase (Shendelman et al., 2004; Taira et al., 2004; Xu et al., 2005; Chen et al., 2010; Richarme et al., 2015). *DJ1* was also shown to interact with other PD-linked genes, such as *PINK1* and α-synuclein, which have been reported to cause PD through mitochondrial dysfunction (Beilina et al., 2005; Dev et al., 2003). These interactions imply that DJ-1 might function as an indirect regulator in PD. Nevertheless, the possible role of DJ-1, which is implied in the pathology of PD, remains poorly understood. In addition, the subcellular localization of DJ-1 has been very controversial, because the cytoplasm, nucleus and mitochondria have all been proposed as its site of residence in the cell (Bonifati et al., 2003; Canet-Avilés et al., 2004; Zhang et al., 2005; Junn et al., 2009).

The *Dictyostelium* mitochondrial disease model is well established and features characteristic and reproducible phenotypic outcomes that in most cases depend on chronic hyperactivity of the energy-sensing protein kinase AMPK (Francione et al., 2011). These aberrant phenotypes include impaired growth and phototaxis and abnormal morphogenesis. If loss of DJ-1 function causes pathology by a mechanism involving impaired mitochondrial function, it should phenocopy the effects of mitochondrial disease. We show here that this is not the case in the *Dictyostelium* model. Instead, *Dictyostelium* DJ-1 is localized in the cytoplasm, where its loss causes cytopathological defects in two endocytic pathways, phagocytosis and macropinocytosis, that are unaffected by mitochondrial dysfunction. In support of this, we use Seahorse respirometry on intact cells to show that knockdown of DJ-1 causes a small but significant activation of mitochondrial respiratory function, whereas overexpression inhibits mitochondrial activity.

## RESULTS

### A single homologue of human DJ-1 was identified in the *D. discoideum* proteome

**Fig. 1.**
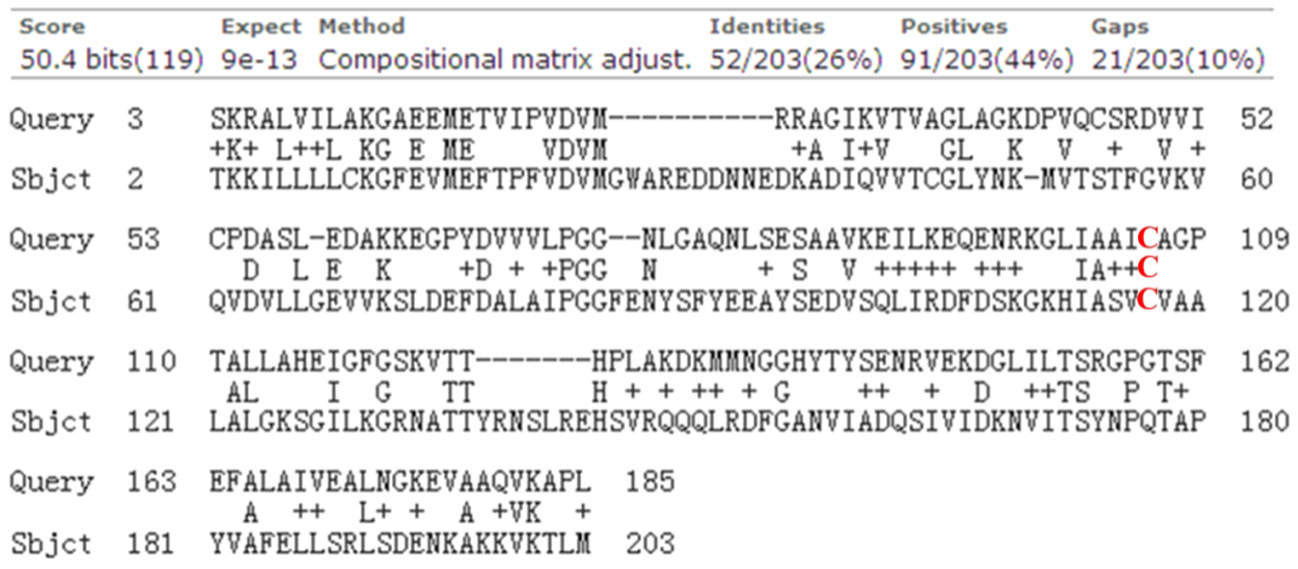
**BLAST sequence alignment using the canonical *Homosapiens* DJ-1 amino acid sequence as the query to search the predicted *D. discoideum* proteome.** The DJ-1 protein in *H. sapiens* is 189 amino acids long, encoded by 23,629 bp with seven exons and six introns (Bonifati et al., 2003), whereas in *D. discoideum*, it contains 205 amino acids, encoded by a gene of only 618 bp with no introns. C106 (highlighted in red) in DJ-1 from *H. sapiens* is a very active and conserved site, which converts to cysteine-sulfinic acid in response to oxidative stress (Canet-Avilés et al., 2004). This residue appears at amino acid 117 in the *D. discoideum* protein.

The protein sequence of human DJ-1 was used at DictyBase with a Basic Local Alignment Search Tool (BLAST) search, and a single homologous protein encoded in the *D. discoideum* genome was identified. The local sequence alignment is shown in Fig. 1.

### *Dictyostelium* DJ-1 is localized in the cytoplasm, not in the mitochondria

The subcellular localization of DJ-1 in mammals is controversial, with conflicting reports of mitochondrial localization (Zhang et al., 2005; Canet-Avlés et al., 2004). To examine the subcellular localization of *Dictyostelium* DJ-1, we first made *in silico* predictions using Web-based software, including MitoProt II, Helical Wheel and Predotar. The results showed that DJ-1 does not have a characteristic mitochondrial targeting signal in the form of a positively charged amphipathic helix at its N-terminus and is unlikely to be localized to the mitochondria, while Predotar predicted that an endoplasmic reticulum (ER) location might be possible (Fig. S1 and Table S1).

**Fig. 2.**
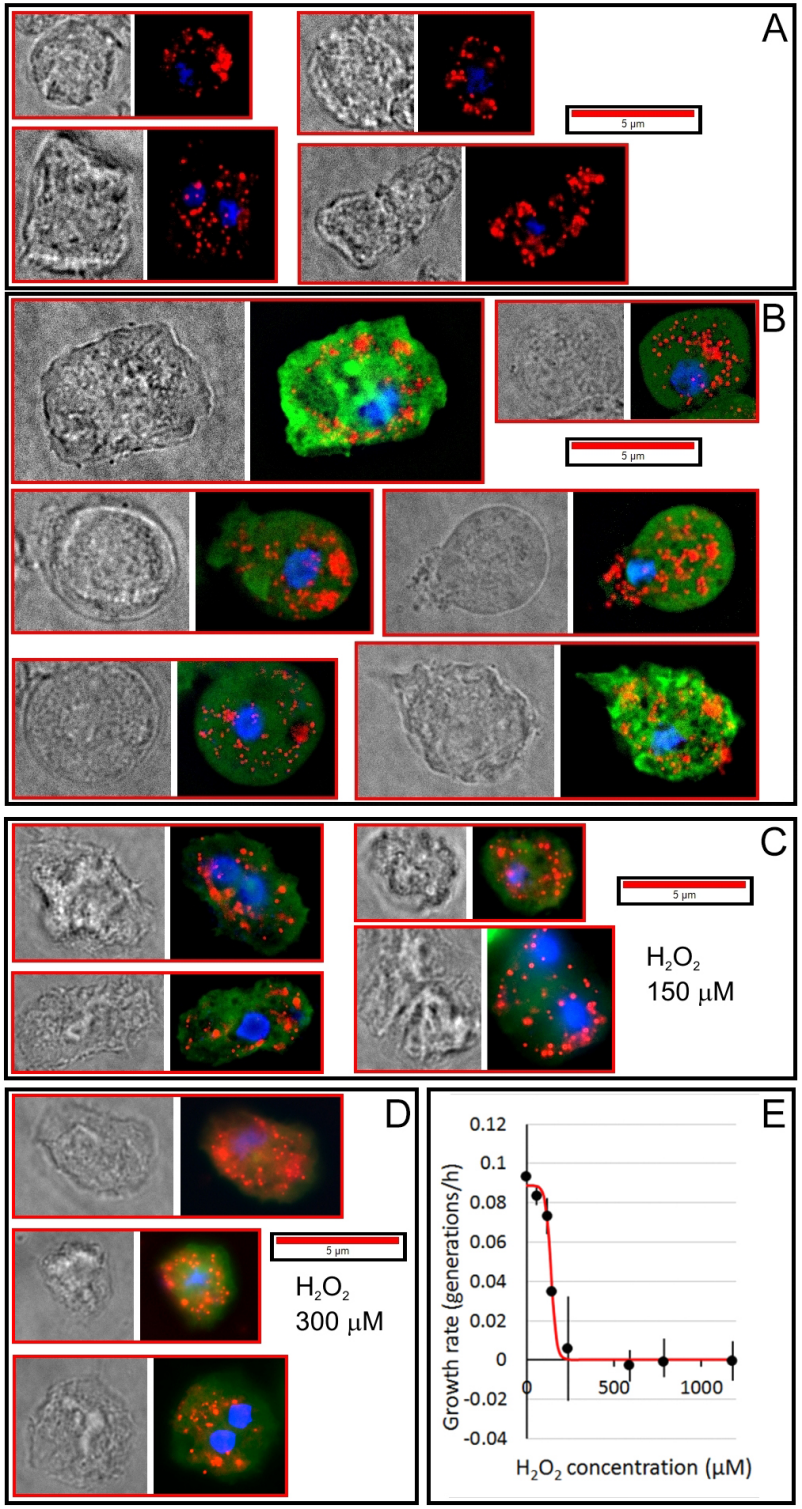
**The subcellular localization of DJ-1 in *D. discoideum*.** Phase contrast and deconvolution fluorescence images of fixed (A) parental and (B-D) DJ-1:GFP-expressing *D. discoideum* cells. Red fluorescence is Mitotracker Red staining of mitochondria. Green fluorescence is DJ-I:GFP (DJ-1 fused to green fluorescent protein expressed in strain HPF1246, a stable transformant of parental strain AX2). Blue fluorescence (DAPI staining) shows the nuclei. There was no green GFP fluorescence in the parental, untransformed strain. In the transformed strain, the DJ-1:GFP fusion protein was found throughout the cell in the cytoplasm, with no enrichment in the mitochondria. Similar results were obtained with Mitotracker Red-stained live cells (Fig. S3) and with traditional epifluorescence microscopy of fixed cells using an anti-GFP antibody to stain the DJ-1:GFP (Fig. S4). No change in the localization was observed after exposure of the cells to H_2_O_2_ for 24 h at concentrations shown to be inhibitory for growth (E), using either fixed (C,D) or live (Fig. S6) cells.

To determine whether the prediction of a nonmitochondrial location for *Dictyostelium* DJ-1 was correct, we created *D. discoideum* cell lines expressing a DJ-1:GFP fusion protein and examined the cells by deconvolution fluorescence microscopy. Fig. 2 shows that DJ-1 was predominantly cytoplasmic, with no evidence of localization in the mitochondria. This was true both in fixed cells (Fig. 2) and in live cells (Fig. S3) and was confirmed by immunofluorescence microscopy of fixed cells using an anti-GFP antibody to detect the GFP (Fig. S4). A western blot separately confirmed that the DJ-1:GFP fusion protein is expressed and has the expected molecular mass in transformants containing the fusion protein construct (Fig. S5).

To determine whether the cytoplasmic localization of the fusion protein was affected by oxidative stress, we grew cells for 24 h in the presence of inhibitory concentrations of H_2_O_2_ (Fig. 2; Fig. S6). The effective inhibitory concentrations were determined by measuring growth rates in these conditions at H_2_O_2_ concentrations ranging up to ∼1.2 mM (Fig. 2E). The concentration giving 50% of maximal inhibition (IC_50_) was found to be 150 µM, and growth was almost completely prevented at 300 µM. The cytoplasmic localization of DJ-1:GFP was unchanged by exposure of the cells to either of these concentrations (Fig. 2C,D).

### Generation of *Dictyostelium* transformants with altered DJ-1 expression

**Fig. 3.**
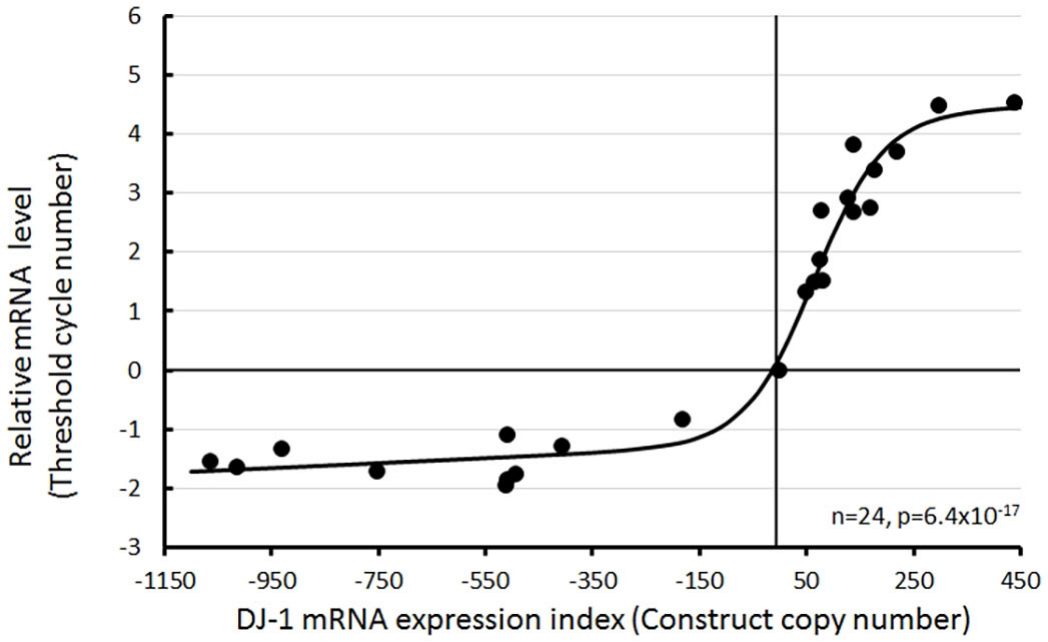
**Expression levels of DJ-1 correlate with the number of copies of the DJ-1 constructs.** Expression levels of DJ-1 versus the number of copies of the antisense inhibition construct, pPROF688, or the overexpression construct, pPROF690. The copy numbers were determined from three independent qPCR experiments, each of which included duplicate measurements. The relative mRNA expression levels (in RT-PCR cycle numbers) for DJ-1 were determined from three independent qRT-PCR, each of which involved duplicate measurements. The values were normalized (by subtraction) against those for the reference gene filamin in each strain before normalizing against AX2 (by subtraction). In accordance with previously established convention, the construct copy numbers were used as an expression index, with overexpression construct copy numbers assigned positive values and antisense construct copy numbers assigned negative numbers (because antisense inhibition reduces expression of the endogenous gene; Bokko et al., 2007). In this and other figures, the parental strain, AX2, is plotted at 0 copies (of both expression constructs). The mRNA expression levels in AX2 are those of the endogeneous *DJ1* gene against which the data were normalized. The regression line was fitted to a modified hyperbolic tangent function by least squares and was highly significant (*F* test).

To study the roles of DJ-1 in otherwise healthy cells, we created *Dictyostelium* transformants with altered levels of DJ-1 expression. Antisense-inhibition (pPROF688) and overexpression constructs (pPROF690) were created and transformed into the parental *D. discoideum* strain AX2 (see Fig. S2 for maps of the plasmid constructs). The plasmid constructs in transformants undergo rolling circle replication to generate multiple copies of the construct, which are integrated randomly into the *D. discoideum* genome by recombination (Barth et al., 1998). This generates stable transformants containing various numbers of copies of the construct, so that each transformant expresses the gene at a different level. The number of copies of the inserted constructs was determined by qPCR, and relative expression levels were measured using qRT-PCR. The relationships between the DJ-1 expression levels and the copy numbers of the antisense and overexpression constructs (Fig. 3) were determined. The antisense inhibition construct reduced DJ-1 mRNA expression by up to about threefold (change of 1.65 qPCR threshold cycles), whereas overexpression resulted in as much as a 23-fold increase in expression (change of 4.5 qPCR threshold cycles) compared with AX2. Thus, the total range in expression levels was nominally about 70-fold from the greatest level of knockdown to the highest level of overexpression.

This approach of combining knockdown and overexpression with analysis of the copy number dependence of the outcomes offers several advantages. It is simple, reliable, lends itself to the study of essential genes and excludes the possibility of phenotypes being caused by unknown genetic events elsewhere in the genome, because such events are random, affect any given phenotype with low probability and are different in every transformant and uncorrelated with construct copy number. Furthermore, regression analysis of the phenotypic outcomes in multiple transformants over a range of copy numbers provides a more sensitive means of reliably detecting abnormalities than can be obtained in the study of one (or two) knockout mutants (which might also contain additional, unknown genetic alterations). In this case, we did not attempt isolation of a knockout strain because of the expectation that it could be lethal.

### Cytoplasmic DJ-1 is needed for normal rates of phagocytosis and micropinocytosis, and reduced levels do not phenocopy mitochondrial dysfunction

To determine the effects of changing the levels of DJ-1 expression, the *D. discoideum* transformants expressing various levels of DJ-1 were analysed phenotypically. If PD cytopathology accompanying DJ-1 deficiency is caused by mitochondrial dysfunction, it should phenocopy the outcomes of mitochondrial disease in *D. discoideum*; these include slow growth, impaired slug phototaxis and defective fruiting body morphology, but no changes in phagocytosis or pinocytosis (except in some isolated respiratory complex deficiencies; Francione et al., 2011; Annesley et al., 2014).

#### Phototaxis was unaffected by altered levels of DJ-1

*D. discoideum* slugs are able to sense light sensitively and move towards it with great accuracy (Fisher, 2001). This enables the slug to migrate to the surface of the soil, which is an optimal location for culmination into a fruiting body and spore dispersal. The photosensory pathway was shown by Wilczynska et al. (1997), Kotsifas et al. (2002), Torija et al. (2006) and others to be sensitive to impaired mitochondrial function. The resulting defect in phototaxis is mediated by chronically hyperactive AMP-activated protein kinase (AMPK), an energy-sensing protein kinase involved in homeostatic regulation of mitochondrial ATP production (Bokko et al., 2007).

**Fig. 4.**
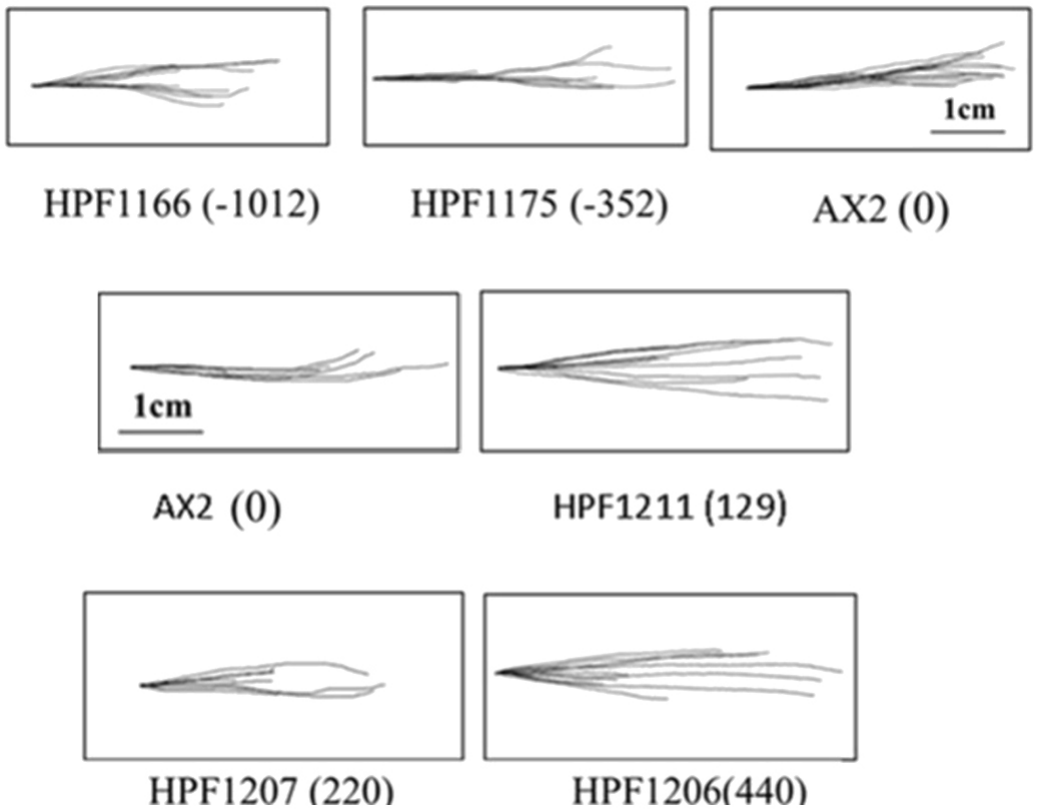
**Phototaxis by slugs of *D. discoideum* DJ-1 transformants with altered DJ-1 expression.** The slug trails of AX2 and DJ-1 transformants are shown migrating to the light source, which is to the right of the figures. HPF1175 and HPF1166 are antisense-inhibited transformants, and the negative numbers in parentheses are the copy numbers of the antisense construct pPROF688, with the negative numbers being used by convention to indicate the resulting reduction in expression. HPF1211, HPF1207 and HPF1206 are DJ-1 overexpression transformants, and the numbers in parentheses are the copy numbers of the overexpression construct pPROF690.

Irrespective of the copy number, phototaxis by slugs of the DJ-1 antisense-inhibited and overexpression transformants displayed highly accurate phototaxis resembling that of the wild type, as shown in Fig. 4. This is different from the features displayed by *D. discoideum* strains with mitochondrial dysfunction, which show disoriented phototaxis, and suggests that DJ-1 does not play a role in photosensory signal transduction and that its reduction does not cause mitochondrial dysfunction in normal conditions.

#### DJ-1 positively regulates growth on bacterial lawns and phagocytosis in *D. discoideum*

**Fig. 5.**
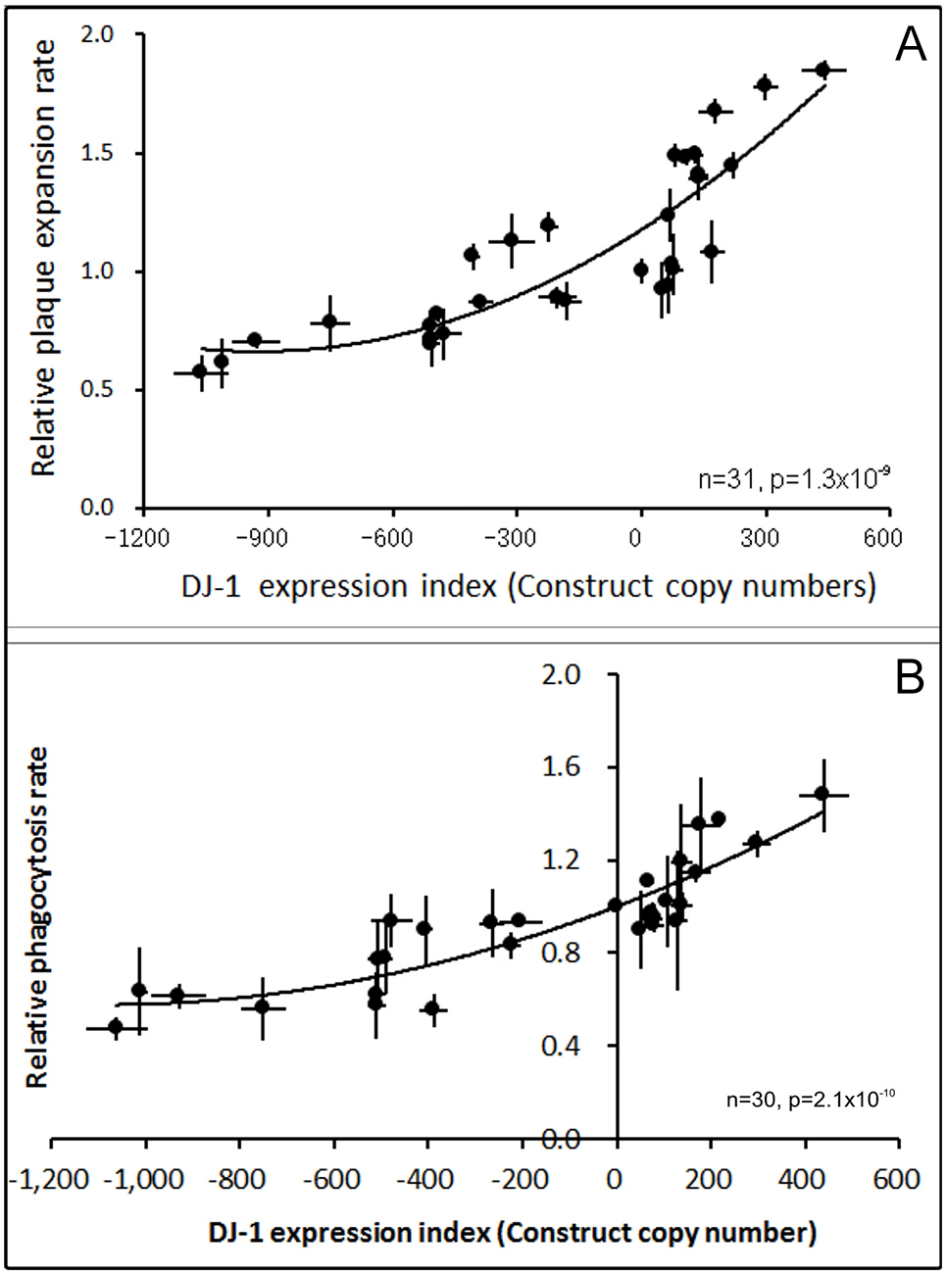
**DJ-1 upregulates growth on bacterial lawns and phagocytosis.** The plaque expansion rates on *E. coli* B2 lawns for the transformants were measured and normalized against the wild-type parent, AX2, then plotted against the DJ-1 expression index. Each point (+s.e.m.) represents the mean from four independent experiments, each of which involved duplicate assays. The regression was fitted by least squares to a quadratic polynomial and was highly significant (*F* test). The phagocytosis rates of the wild-type AX2 and the transformants with altered DJ-1 expression were measured and normalized against AX2. Each point represents the mean from three independent experiments, each of which included duplicate measurements. The regression was highly significant (*F* test). Error bars are s.e.m.

Mitochondrially diseased *Dictyostelium* strains showed decreased growth on bacterial lawns. To investigate whether DJ-1 is involved in regulating growth and whether it might be involved in mitochondrial dysfunction, the DJ-1 transformants were analysed for their ability to grow on normal agar plates containing a lawn of *Escherichia* *coli* B2. The rate of plaque expansion was calculated and compared with wild-type AX2. Fig. 5A shows that DJ-1 knockdown decreases plaque expansion rates, whereas overexpression increases plaque expansion rates. This elevated rate of plaque expansion was also exhibited by cells expressing the DJ-1:GFP fusion protein (strain HPF1246), which grew 30% faster than wild type on *E. coli* B2 lawns (*P*=1.8×10^−6^, multiple linear regression of plaque diameters versus time).

The rate of *D. discoideum* plaque expansion on bacterial lawns is controlled by phagocytosis rate, growth rate and motility of the amoebae. To determine whether the increased rate of plaque expansion on bacterial lawns could be explained by increased rates of phagocytosis, we measured the rate of uptake of fluorescently labelled bacterial prey. Fig. 5B shows that knockdown of DJ-1 expression reduced phagocytosis rates, whereas increased expression resulted in greater phagocytosis rates. The magnitude of the effect across the whole range of DJ-1 expression levels was two- to threefold, similar to the effect on plaque expansion rates. Not suprisingly, the phagocytosis and plaque expansion rates were also correlated with one another (not shown). These results suggest that the changes in plaque expansion rates were attributable to effects of DJ-1 levels on the phagocytosis rates. This is unlike most forms of mitochondrial disease in *Dictyostelium*, in which, except for some isolated Complex I-specific defects, growth on bacterial lawns is impaired but phagocytosis is unaffected (Francione et al., 2011).

#### DJ-1 modestly upregulates axenic growth and pinocytosis

**Fig. 6.**
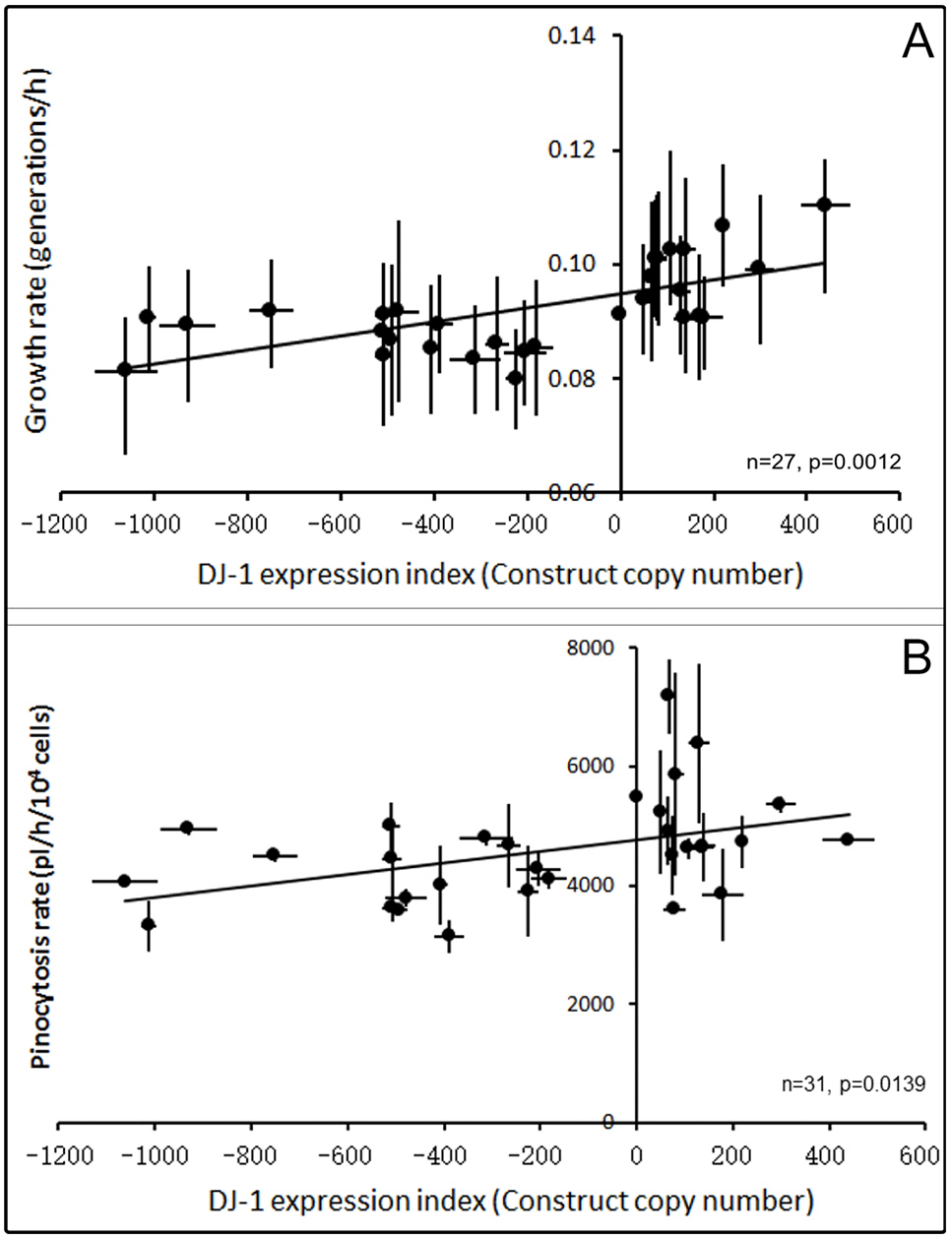
**DJ-1 modestly upregulates growth in liquid medium and pinocytosis.** (A) The growth rate (inverse of the doubling time) during exponential growth of DJ-1 transformants was measured and compared with the parent strain, AX2. The linear regression was highly significant (*F* test). Error bars are the s.e.m. from three independent experiments. (B) AX2 and transformants were grown in low-fluorescence HL-5 medium containing FITC-dextran. The pinocytosis of the transformants increased as the DJ-1 expression levels were increased. The linear regression was statistically significant (*F* test). Error bars are the s.e.m. from three independent experiments.

Laboratory strains of *D. discoideum* are also able to grow axenically, without a bacterial food source, in nutrient broth (HL-5). To determine whether changing the expression of DJ-1 has effects on axenic growth, the generation time of wild-type AX2 and the DJ-1 antisense-inhibited and overexpression transformants was measured. Fig. 6A shows that the growth rate increases (generation time decreases) modestly with increasing DJ-1 expression. Although statistically significant and consistent with the effects on growth on bacterial lawns, this effect is only small, with a 2 h (*∼*20%) decrease in generation time over the *∼*70-fold range in DJ-1 mRNA expression levels between the highest copy numbers of the antisense and overexpression constructs. It is dissimilar in magnitude from the large impairment of growth in axenic medium observed in all mitochondrially diseased *Dictyostelium* strains (Francione et al., 2011).

To determine whether the foregoing changes in growth rates in liquid medium were attributable to changes in the ability of the transformants to ingest nutrients from the liquid medium, the rates of pinocytosis were measured. The pinocytosis rates did correlate weakly with the expression of DJ-1 (Fig. 6B), changing by *∼*20% across the full range of *DJ1* gene expression. This suggests that the slight effects on the generation time in liquid medium were a result of similar modest effects on the rate of pinocytosis. Except for some Complex I-specific defects, mitochondrial disease in *Dictyostelium* does not cause impairment of pinocytosis (Francione et al., 2011).

#### DJ-1 is required for normal fruiting body morphology

**Fig. 7.**
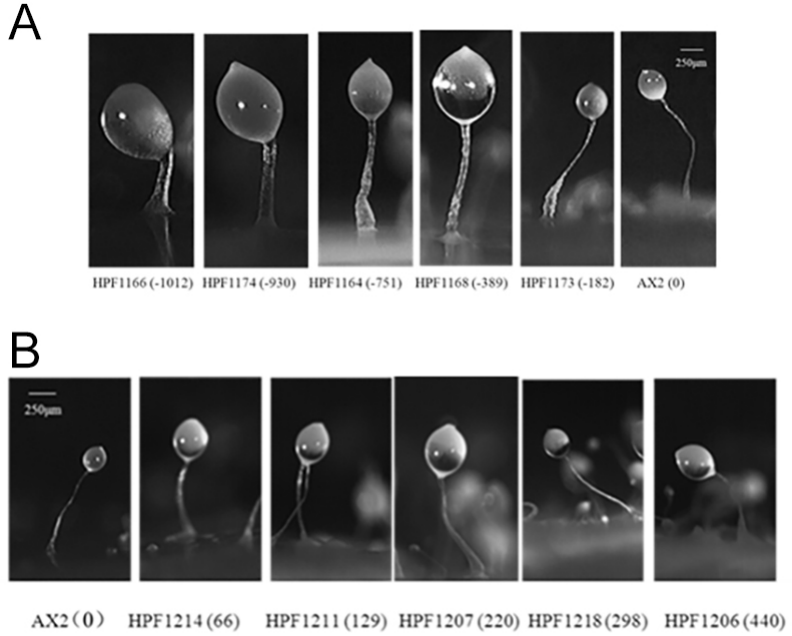
**DJ-1 knockdown causes aberrant *Dictyostelium* morphogenesis.** (A) The fruiting bodies of DJ-1 antisense-inhibited transformants. Strain names are indicated by HPF numbers, and the negative numbers in parentheses represent the copy number of pPROF688 (antisense-inhibition construct). The spore droplets (sori) are enlarged, and the stalks are thicker and shorter in the DJ-1 antisense-inhibited strains. The severity of the defect correlates with the reduction in DJ-1 expression. (B) The fruiting bodies of DJ-1 overexpression transformants. Strain names are indicated by HPF numbers, and the numbers in brackets represent the copy number of pPROF690 (overexpression construct). Wild-type AX2 fruiting bodies contain long, slender stalks, as do the transformants overexpressing DJ-1.

When their bacterial food source is depleted, *D. discoideum* amoebae aggregate and undergo multicellular differentiation, ultimately resulting in the formation of a fruiting body that consists of a sorus containing spores supported by a stalk and basal disc consisting of vacuolated cells (Strmecki et al., 2005). Mitochondrial dysfunction and activation of AMPK in *D. discoideum* results in altered fruiting body morphology, with fewer fruiting bodies that have shorter and thicker stalks (Kotsifas et al., 2002; Bokko et al., 2007). To investigate whether DJ-1 was involved in regulating normal fruiting body morphogenesis, the fruiting bodies of the wild-type AX2 and DJ-1 transformants were observed. A reduction in the expression of DJ-1 resulted in aberrant fruiting bodies with larger sori and shorter, thicker stalks, whereas an increase in the expression of DJ-1 did not result in any alterations when compared with AX2 (Fig. 7). Although this phenotype is present in mitochondrially diseased *Dictyostelium* strains, it is also caused by lysosomal defects in *Dictyostelium* models for Batten disease (Chavan, 2015). The various forms of Batten disease are neurodegenerative disorders caused by mutations in lysosomal or ER proteins, and they produce lysosomal, vesicle trafficking and autophagy abnormalities (Narayan et al., 2006; Siintola et al., 2005; Wavre-Shapton et al., 2015). The short, thick stalks in DJ-1 knockdown strains thus constitute a phenotype which, in isolation, does not allow a distinction to be made between mitochondrial and lysosomal disorders.

### DJ-1 inhibits and its loss activates respiration by functionally normal oxidative phosphorylation complexes

**Fig. 8.**
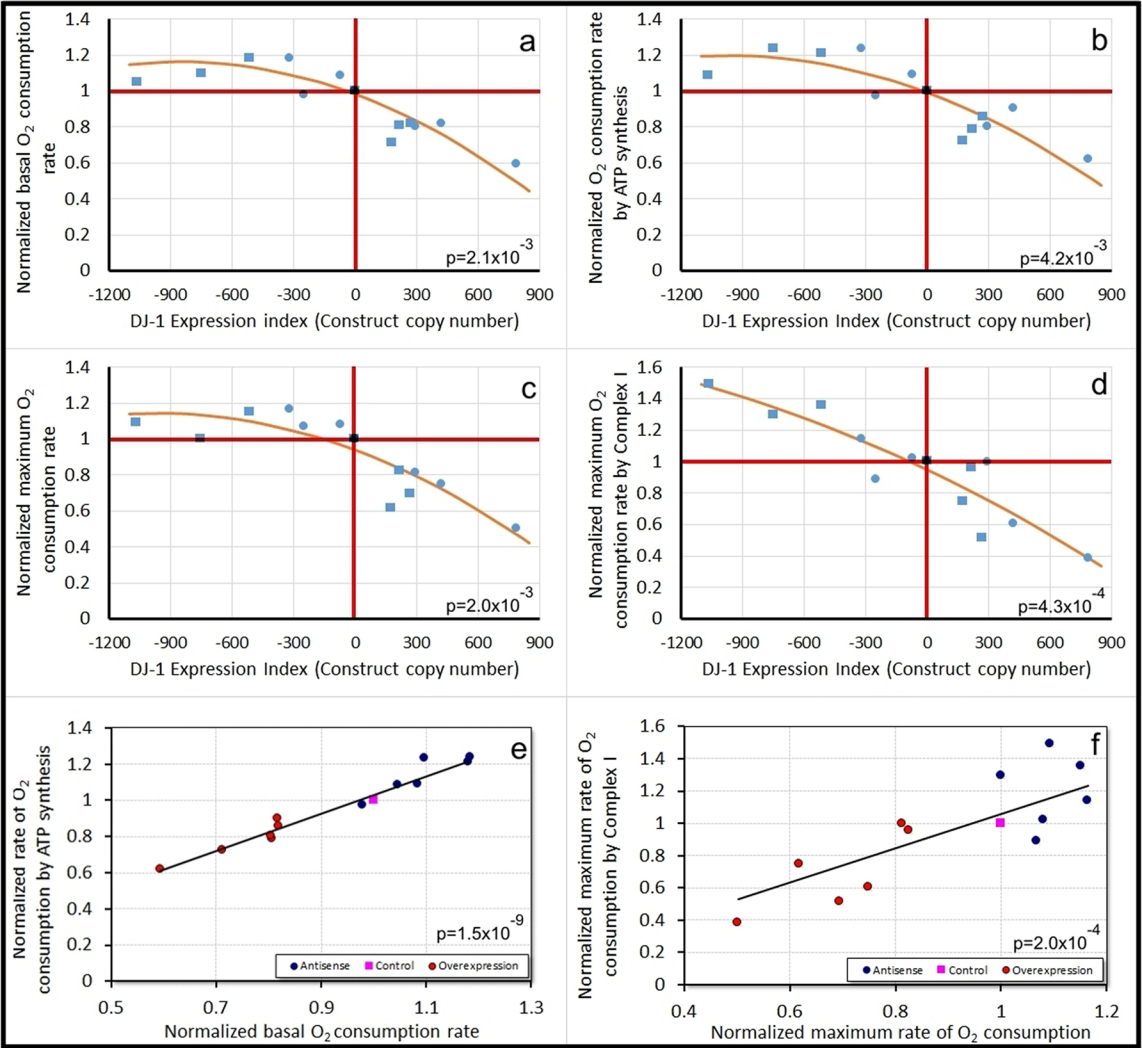
**Mitochondrial respiratory activity is inhibited by increasing DJ-1 expression levels.** Seahorse respirometry was used on intact *Dictyostelium* amoebae (Lay et al., 2016) in two separate series of independent experiments involving two wild-type control strains, AX2 and HPF401. Three cognate, otherwise isogenic DJ-1 antisense-inhibited strains and three cognate, otherwise isogenic DJ-1 overexpressing strains were tested for each of the wild-type control strains (AX2, squares in A-D; HPF401, circles in A-D). The O_2_ consumption rates (OCR) were normalized against the average values for the cognate control strain. Each point represents the average from 2-17 independent experiments on a given strain, with each experiment involving four technical replicates (separate wells in the assay plate). All regressions were highly significant (significance probabilities shown in each panel margin). (A-D) The dependence of key parameters of respiration on the level of expression of DJ-1, as indicated by the construct copy number. Following previously established convention (Bokko et al., 2007), the copy numbers for the antisense inhibition construct are assigned negative values. The basal OCR (A) and OCR attributable to ATP synthesis (B) represent mitochondrial respiration in intact cells. Maximum oxygen consumption rates (C) represent the combined activities of Complexes I and II in carbonyl cyanide 3-chlorophenylhydrazone (CCCP)-uncoupled mitochondria and other cellular oxidases and oxygenases, whereas O_2_ consumption by Complex I (D) represents the contribution of Complex I alone to the maximal OCR. (E,F) The relationship between basal and maximal uncoupled respiration rates and key components thereof. The proportion of basal O_2_ consumption by mitochondrial ATP synthesis, and of maximal O_2_ consumption by Complex I are unaffected by DJ-1 knockdown or overexpression, so that the points in both groups of strains and the control strain all lie on the same regression line through the origin (line slopes not significantly different in multiple regression analysis). If a given respiratory complex were functionally impaired in the DJ-1 antisense-inhibited or overexpression strains, it would contribute a different (smaller) fraction of respiratory O_2_ consumption and so lie on a different regression line. Similar results were obtained with all other assayed components of respiration (Fig. S7). By contrast with the effect of changing DJ-1 expression levels, there was no effect on mitochondrial respiration of the expression of aequorin, a Ca^2+^-responsive luminescent protein that will allow assay of the impact of DJ-1 on Ca^2+^ signalling in future studies (Fig. S8).

The cytosolic location of DJ-1 and the failure of DJ-1 knockdown faithfully to phenocopy mitochondrial dysfunction suggest that loss of function of this protein can cause cytopathology without causing damage to mitochondrial respiratory functions. To verify whether this is so, we used Seahorse respirometry (Lay et al., 2016) to assay mitochondrial respiratory activity directly in the parental AX2 strain and in DJ-1 antisense-inhibited and overexpressing strains. We found that reduced levels of DJ-1 expression slightly enhanced, whereas overexpression inhibited key parameters of mitochondrial respiratory function, namely the basal O_2_ consumption rate (OCR; Fig. 8A), the O_2_ consumption attributable to respiratory ATP synthesis (Fig. 8B), and the uncoupled respiration rate (Fig. 8C) and Complex I (Fig. 8D) activities. Regression analysis of O_2_ consumption by ATP synthesis versus basal OCR (Fig. 8E), and Complex I activity versus the uncoupled OCR (Fig. 8F) showed that the antisense, control and overexpression strains all fell on the same straight line. This shows that ATP synthase and Complex I are functionally normal, making the same relative contribution to respiration in the antisense and overexpression strains. Similar results were observed (Fig. S7) with all other assayed components of respiration. Thus, changes in the level of expression of DJ-1 affect only the total level of respiratory activity, not the functionality of individual complexes. We conclude that loss of DJ-1 does not impair but activates mitochondrial respiratory function, whereas overexpression causes clear reductions in all mitochondrial respiratory activity. These effects are not consistent with a protective role for DJ-1 in the mitochondria and must be indirect because the protein is not localized to the mitochondria.

## DISCUSSION

Mutations of DJ-1 (14 kb deletion and L166P) have been found to be associated with autosomal recessive early-onset PD, which is considered to result from loss of function of the DJ-1 protein (Bonifati et al., 2003). Accordingly, DJ-1 is believed to play a normally protective role in the cell by functioning as a molecular chaperone, antioxidant, transcriptional regulator, protease and/or protein deglycase (Bonifati et al., 2003; Taira et al., 2004; Shendelman et al., 2004; Paterna et al., 2007; Clements et al., 2006; Olzmann et al., 2004). Mitochondrial dysfunction has been implicated in the pathogenesis of PD, and DJ-1 has been suggested to have a protective role in mitochondrial function (Larsen et al., 2011). However, Giaime et al. (2012) found that DJ-1 knockout does not impair mitochondrial respiratory function in primary mouse embryonic fibroblasts.

To exert a putative protective role in the mitochondria, DJ-1 needs to be localized to the mitochondria. Conflicting data in this regard have been presented in the literature. Zhang et al. (2005) found that DJ-1 localized to the mitochondrial matrix and inter-membrane space in mouse brain tissues and human neuroblastoma cells. However, Canet-Avlés et al. (2004) found that DJ-1 has very little specific localization to mitochondria in M17 human neuroblastoma cells, although treatment with paraquat (pQ^2+^) created an oxidative stress and led to the translocation of DJ-1 to the mitochondria. In the present study, three *in silico* bioinformatics tools (MitoProt II, Predotar and Helical Wheel) were used to predict the subcellular localization of DJ-1. The results in Fig. S1 and Table S1 suggested little likelihood that DJ-1 normally has a mitochondrial location. The helical wheel plot of DJ-1 did not exhibit the characteristic positively charged amphiphilic α-helix at the N-terminus that constitutes the mitochondrial targeting signal for most mitochondrial proteins (Fig. S1B). In addition, mitochondrial proteins usually contain an arginine residue, which can be recognized by mitochondrial processing peptidases and mitochondrial intermediate peptidases (Endo and Kohda, 2002; Mills et al., 2008). These enzymes remove the leader peptide upon import of the protein into the mitochondrial matrix, to produce the mature mitochondrial protein. DJ-1 does contain an arginine (R29) near the N-terminus, but the surrounding sequence does not conform to the consensus target site for mitochondrial processing peptidases (Lee et al., 2003).

Fluorescence microscopy using a GFP-tagged DJ-1 (Fig. 2; Figs S3, S4, S6) supported this, by showing that DJ-1 is cytoplasmic both in unstressed and in oxidatively stressed cells. Like the wild-type protein, expression of the DJ-1:GFP fusion protein caused accelerated growth on a bacterial lawn. This suggests that the GFP-tagged protein is functionally normal and that its subcellular location thus reflects that of the wild-type protein.

Although DJ-1 is localized in the cytoplasm, its loss could impair mitochondrial function indirectly. If so, mitochondrial respiration should be impaired by DJ-1 loss (knockdown) and enhanced by its overexpression. When we measured mitochondrial respiratory activity directly in intact, living *Dictyostelium* cells, we found that the reverse was true; DJ-1 knockdown resulted in a small, but statistically significant increase in mitochondrial respiration, whereas overexpression caused a reduction in the key parameters of mitochondrial respiratory activity. It is of interest that similar respirometric measurements in intact human cells recently revealed enhanced mitochondrial respiration both in lymphoblasts from idiopathic PD patients (Annesley et al., 2016) and in fibroblasts from Parkin-null genetic PD patients (Haylett et al., 2016).

*D. discoideum* is an established mitochondrial disease model, with highly predictable and reproducible disease phenotypes. If the loss of DJ-1 causes mitochondrial respiratory dysfunction, the *D. discoideum* model of PD created by DJ-1 knockdown would phenocopy the well-established outcomes of mitochondrial disease. The results reported here show that this does not happen. Firstly, the phototaxis defect that is observed in all mitochondrially diseased *Dictyostelium* strains (Wilczynska et al., 1997; Kotsifas et al., 2002; Bokko et al., 2007; Francione et al., 2009) was not present in DJ-1 knockdown strains. Secondly, the DJ-1 knockdown strains exhibited growth defects associated with commensurately impaired rates of endocytosis, a feature not present in mitochondrially diseased strains, with the exception of some isolated Complex I-specific defects (Kotsifas et al., 2002; Torija et al., 2006; Bokko et al., 2007). Thirdly, the growth defects observed in mitochondrially diseased strains are equally severe whether the cells are grown on bacterial lawns or in liquid medium. This was not the case with the DJ-1 knockdown strains, in which the impairment of phagocytosis and growth on bacterial lawns was much more severe than the slight effects on macropinocytosis and growth in liquid medium. These results showing differential dependence of different types of endocytosis on DJ-1 are reminiscent of those of Kim et al. (2013), who reported that astrocytes of DJ-1 knockout mice exhibit impaired lipid raft-dependent but not clathrin-dependent endocytosis. DJ-1 was associated with lipid rafts, where it was required for CD14-dependent endocytosis of the pattern recognition receptors TLR3 (recognizing double-stranded RNA) and TLR4 (recognizing bacterial lipopolysaccharide). These observations and the role of DJ-1 in phagocytosis in *Dictyostelium* are consistent with the reported role of lipid rafts in phagocytosis (Nagao et al., 2011).

Under nutrient deprivation *Dictyostelium* amoebae differentiate, chemotactically aggregate and undergo a transition to a multicellular differentiation programme that ultimately results in formation of a fruiting body that consists of a sorus containing spores and a long slender stalk and basal disc that contain dead, vacuolated cells, the end product of a form of autophagic cell death. This process, which is affected by defects both in mitochondrial respiration and in lysosomal function, results in more cells entering the stalk differentiation rather than the spore differentiation pathway (Bokko et al., 2007; Francione et al., 2011; Chavan, 2015). The result is fruiting bodies with short, thick stalks, as we observed in this work in our DJ-1 antisense-inhibited transformants (Fig. 7A). This suggests that DJ-1 plays a role in preventing cells from differentiating into stalk cells, possibly through preventing autophagic cell death.

The results presented here show that rather than impairing mitochondrial respiration, the loss or reduction of function of DJ-1 in Parkinson's disease cells produces defects in particular endocytic pathways, downstream vesicle trafficking and related processes. At least 17 other Parkinson's disease-related proteins also play normal and cytopathological roles in endolysosomal trafficking, and another six are associated with autophagic trafficking to the lysosome of defective organelles and protein aggregates (Abeliovich and Gitler, 2016). The outcome of reduced DJ-1 expression in *Dictyostelium* is chronic nutritional stress and slow growth, accompanied by elevated autophagic cell death in the form of short, thick stalks.

It has been suggested from work on cell lines and animal models that DJ-1 plays a protective role in cells, shielding them from the adverse downstream consequences of oxidative and other stresses (Bonifati et al., 2003; Taira et al., 2004; Inden et al., 2006; Paterna et al., 2007; Hwang et al., 2013). Although we did not explicitly stress the cells in most experiments reported in this paper, even healthy cells experience background levels of a variety of stresses including oxidative stress, unfolded proteins, DNA damage and the like. However, to determine whether DJ-1 translocates to the mitochondria under oxidative stress, we exposed growing cells to inhibitory concentrations of H_2_O_2_ for 24 h. The cytoplasmic localization of the DJ-1:GFP fusion protein was unaffected by this treatment. Given that DJ-1 has protective molecular chaperone, protease and protein deglycase activities, it could exert these functions normally in nonmitochondrial locations to protect endocytic and associated vesicle trafficking pathways from the cytopathological consequences of oxidative and other stresses. Our results do not allow us to distinguish between positive regulatory or protective roles for DJ-1 in endocytosis, but they do reveal that the loss of DJ-1 function can cause cytopathological consequences in particular endocytic pathways that are not associated with impaired mitochondrial function. It will be of interest in future work to determine whether these roles of DJ-1 role depend on its proteolytic, deglycation or chaperone functions.

The abnormal phagocytosis, growth and morphogenesis in DJ-1 knockdown strains are similar to those caused by high levels of ectopic expression in *Dictyostelium* of wild-type and mutant forms of another PD-associated protein, human α-synuclein (Fernando, 2012). As in the work described in this paper, the α-synuclein was not localized in the mitochondria but in the cytosol (albeit concentrated in the cortical regions), and the pattern of phenotypes was unlike that observed in mitochondrially diseased strains. In mammalian cells, mutant α-synuclein (A30P) expression impairs proteasomal activity, possibly via interaction with a subunit of proteasome regulatory complexes (Ghee et al., 2000; Tanaka et al., 2001). It will be of interest in future experiments to study potential interactions between DJ-1 and α-synuclein in the *Dictyostelium* cytosol.

The existence of nonmitochondrial endocytic defects resulting from reduced DJ-1 expression suggests that the cytopathological mechanisms underlying Parkinson's disease include processes not involving mitochondrial dysfunction. However, this does not exclude the possibility of additional pathological mechanisms, particularly in oxidatively stressed cells. It will be valuable to examine this possibility in future work.

## MATERIALS AND METHODS

### Bioinformatic analysis of *Dictyostelium* DJ-1

A BLAST at DictyBase (the international dictyostelid genomics resource at http://dictybase.org; Basu et al., 2013) was used to identify the homologue of human DJ-1 in the *D. discoideum* proteome using the protein sequence of human DJ-1. The programs MitoProt II, Predotar and Helical Wheel were used to predict the possibility of subcellular localization of DJ-1.

### Plasmid constructs

DJ-1 antisense construct pPROF688 and DJ-1 sense construct pPROF690 were created by subcloning a fragment of the *DJ1* gene (75-479 bp) and the whole *DJ1* gene into the vector pDNeo2 (Witke et al., 1987) and the vector pPROF267 (generated by S.J.A., unpublished data) by replacing the GFP cassette in pA15GFP with the Tet cassette from pPROF74 and additional restriction enzymes sites included for cloning purposes. The construct pPROF693 was generated by insertion of the *DJ1* gene in frame into pA15GFP for expression of DJ-1:GFP fusion protein.

### *D. discoideum* strains and culture conditions

All experiments were conducted with *D. discoideum* parental strain AX2 and transformants derived from it (Wilczynska et al., 1997). Strains HPF1164-HPF1179 carried multiple copies of the DJ-1 antisense inhibition construct pPROF688, strains HPF1206-HPF1219 carried multiple copies of the DJ-1 sense construct pPROF690, and strains HPF1245-HPF1246 contained the construct pPROF693, which enable expression of DJ-1:GFP. In addition to AX2 itself, we also used HPF401 as a wild-type control in some experiments, when appropriate. HPF401 is an AX2 transformant containing pPROF120 (Nebl and Fisher, 1997), an aequorin-expression construct that enables recombinant aequorin-based measurement of cytosolic Ca^2+^ in *Dictyostelium*, but has no effect on any of the phenotypes assayed in this work. DJ-1 antisense and overexpression strains used in this work that also contain pPROF120 were HPF1274-HPF1276 (antisense construct pPROF688) and HPF1277-HPF1279 (overexpression construct pPROF690).

On solid medium, *D. discoideum* cultures were maintained at 21°C with *Klebsiella aerogenes* as a food source on Standard Medium (SM) agar plates containing 20 μg ml^−1^ G418. *D. discoideum* cells were also maintained in HL-5 liquid media supplemented with 50 μg streptomycin ml^−1^, 10 μg tetracycline ml^−1^ and 100 μg ampicillin ml^−1^. All antibiotics were removed when growing cells for and conducting all phenotypic assays. This ensures the stability of the transformants, as they are not maintained for long periods without selection, while also removing the possibility of an effect of the antibiotics themselves on the resulting phenotypes. Stock cultures for all strains were kept frozen in DMSO.

### Isolation and molecular manipulation of DNA and RNA

#### DNA and RNA isolation

Plasmid DNA isolation was adapted from Birnboim and Doly (1979) and Birnboim (1983); and the PureLinkTM HiPure Plasmid Filter Purification Kit and the PureLinkTM HiPure Precipitator Module (Invitrogen) were used for the large-scale extraction of plasmid DNA. DNAzol (Molecular Research Center) and TRIzol reagent (Gibco BRL) were used to isolate genomic DNA and RNA from *D. discoideum* transformants according to the instructions of the manufacturer. The CsCl isolation method was used for the large-scale isolation of *D. discoideum* genomic DNA as described in DictyBase (http://dictybase.org).

#### DNA and RNA molecular manipulation

General gene cloning and sequence analysis were conducted as described previously (Wilczynska et al., 1997; Kotsifas et al., 2002). Fragments of interest were amplified using gene-specific primers containing added restriction sites at the 5′ end for cloning and subcloning purposes. Constructs were verified by restriction endonuclease digestion and DNA sequencing [Australian Genome Research Facility (AGRF), Melbourne]. Sequence alignment and database searches were performed using software BioEdit and Web-based software at ExPASy (http://www.expasy.org).

#### Polymerase chain reaction

A 405 bp *DJ1* gene encoding the DJ-1 antisense fragment was amplified, cloned into pUC18 and subcloned into pDNeo2 vector with primers DAF (5′-GCGAATTCGAGCTCGGGTTGGGCTAGAGAGG-3′) and DAR (5′-GCGAATTCGGATCCGCGATAACGTTTGCACCG-3′). The full length of DJ-1 was amplified, cloned into pUC18 and subcloned into pPROF267 with primers DOEF (5′-GCGAATTCATCGATATGACCAAAAAAATATTATTATTATTATGTAAAGG-3′) and DOER (5′-GCGAATTCCTCGAGTTAAAAACCCATTAAAGTTTTTACTTTTTTAGC-3′). The *DJ1* gene without the stop codon was amplified and cloned into the pUC18 vector and subcloned into pA15GFP for expression of DJ-1:GFP with the same forward primer as DOEF and DGFPR (5′-GCGAATTCATCGATAAAACCCATTAAAGTTTTTACTTTTTTAGC-3′).

##### Quantitative PCR (qPCR)

The construct copy numbers in *D. discoideum* transformants were measured by quantitative PCR using iQ SYBR Green Supermix as instructed by the manufacturer (Bio-Rad). The gDNA of *D. discoideum* AX2 was used to create a standard curve for estimation of the quantity of gDNA. The filamin gene (Annesley et al., 2007) was used as a reference housekeeping gene, and primers were designed using software Primerquest to amplify a 100 bp fragment from the endogenous filamin. The reaction was performed using an iCycler IQ Multicolor Real-Time PCR Detection System (Bio-Rad). The calculations of copy numbers for each gene were based on comparison of their average Ct values (threshold cycle) with those for filamin (control for gDNA concentration) in unknown and parental AX2 (control for gene's quantity in chromosomal DNA). The linear relationship of the logarithm of the DNA amount (Starting Quantity) and its Ct value enables the calculation of gene copy numbers in transformants.

##### Quantitative one-time reverse transcriptase-PCR (qRT-PCR)

The RNA was quantitated by iScriptTM One-Step RT-PCR Kit. The RNA from all strains was extracted and treated with DNase I before it was mixed with SYBR Green RT-PCR reaction mix, primers and iScript reverse transcriptase. Two transformants was chosen randomly for use as negative controls, in which iScript reverse transcriptase was not added. The reaction was carried out in an iCycler IQ Multicolor Real-Time PCR Detection System (Bio-Rad), and the copy numbers of cDNA were calculated as described for qPCR.

### Fluorescence microscopy

Axenically grown cells were allowed to settle onto glass coverslips for 30 min. For immunofluorescence, the HL-5 was replaced with Lo-Flo-HL5 medium for 1 h before staining. The medium was then replaced with 200 nM Mitotracker Red stain in PBS (CMX-Ros; Invitrogen Molecular Probes) and incubated for 45 min to stain the mitochondria. The cells were either washed three times in PBS and examined directly (live cells) or fixed.

For immunofluorescence (Fig. S4), fixation was performed with the addition of 1 ml of 3.7% (v/v) formaldehyde in PBS for 30 min followed by removal of the paraformaldehyde and incubation with prechilled methanol (−20°C) for 5 min to make the cells permeable. The coverslip was then washed twice with PBS before blocking for 1 h, gently shaking in blocking buffer, which was then replaced for overnight incubation with anti-GFP IgG conjugated with Alexa-FluorR488 (Molecular Probes, Invitrogen; 1:500 in blocking buffer). The coverslips were washed three times with PBS, then incubated with 0.1 μg DAPI in PBS for 5 min to stain nucleic acids. Excess stain was removed by washing twice with PBS and once with milliQdH2O.

Otherwise, fixation was performed for 10 min in 300 µl 4% paraformaldehyde in PBS followed by 10 min in 4% paraformaldehyde plus 0.2% Triton X-100, then by two washes in PBS and 7 min incubation in 0.2 M glycine in PBS. The coverslip was then washed three times in PBS, once in dH_2_O and finally mounted onto a slide using 5 µl of mounting media with DAPI (Duolink).

The cells were observed with an Olympus BX 61TRF microscope. The digital images were captured with an Olympus DP80 camera. For deconvolution microscopy, the image was deconvolved after background subtraction, using Olympus cellSens Dimension 1.16 software. Deconvolution removes out-of-focus light to produce a result similar to laser confocal microscopy.

To examine the effect of oxidative stress, the same procedures were followed, except that the cells were exposed to inhibitory concentrations of H_2_O_2_ (150 and 300 µM) for 24 h in axenic medium before and during the Mitotracker Red staining.

### Transformation of *D. discoideum* and phenotypic analysis

#### Transformation of *Dictyostelium*

All transformants were obtained using the Ca(PO_4_)_2_/DNA coprecipitation method developed by Nellen et al. (1984) with minor alterations and isolated by growing on *Micrococcus luteus* lawns on SM agar supplemented with 20 μg ml^−1^ G418 described by Wilczynska and Fisher (1994).

#### Phenotypic analysis of *Dictyostelium* strains

##### Plaque expansion

*Dictyostelium* strains were inoculated in the centre of a Normal agar plate containing a lawn of *E. coli* B2, with four replicates for each strain. The plates were incubated at 21°C, and the diameter of the plaque was measured twice each day at ∼8 h intervals for 100 h. The statistics software package ‘R’ was used to calculate and analyse the plaque expansion rate (in mm/h) using linear regression analysis.

##### Phototaxis analysis

The phototaxis assay was performed using the methods developed by Wilczynska and Fisher (1994) and Fisher and Annesley (2006). *D. discoideum* colonies growing on *K. aerogenes* lawns were collected and spotted onto charcoal agar plates. The plates were placed into black polyvinylchloride (PVC) boxes with a 4 mm hole drilled in one side and incubated at 21°C for 24-36 h with the hole facing a lateral light resource. The amoebae aggregated and formed slugs, which migrated over the agar surface leaving a trail of collapsed slime sheath, which was analysed and transferred to PVC discs and stained with 0.3% (v/v) Coomassie Blue R. The stained slug trails were digitized as described by Darcy et al. (1994) using a Summagraphics MM1201 digitizing tablet connected to a Linux workstation.

##### Growth in HL-5 broth

The axenic growth of *D. discoideum* strains was conducted and its growth rate measured as previously described (Bokko et al., 2007). The *Dictyostelium* cells were inoculated into HL-5 liquid medium and incubated at 21°C, shaking at 150 rpm. Cells were counted using a haemocytometer (Scientific Instruments) twice a day at an interval of 7-8 h for >100 h. The programmable software package ‘R’ was used for statistical analysis and calculation of generation time after log-linear regression analysis. In some experiments, to determine the inhibitory effects of oxidative stress on growth, the medium was supplemented with H_2_O_2_ at concentrations in the micromolar range and up to ∼1.2 mM.

##### Morphogenesis

Fruiting body morphology of *D. discoideum* cells was examined as described previously (Kotsifas et al., 2002; Bokko et al., 2007). The *Dictyostelium* strains were streak diluted onto *K. aerogenes* lawn on SM plates and incubated at 21°C for ∼48 h until they underwent multicellular development. The fruiting body morphology was analysed using an Olympus SZ61™ dissecting microscope, and the photographs were taken using an attached Moticam 2300™ camera. A slice of agar containing fruiting bodies was excised for the side view of fruiting body morphology.

##### Phagocytosis

The phagocytosis rate of *Dictyostelium* strains was determined using the prey of *E. coli* cells, which express a fluorescent protein named DSRed (Maselli et al., 2002; Bokko et al., 2007). The *E. coli* DSRed cells were inoculated into Luria broth containing ampicillin (75 μg ml^−1^) and 1 mM IPTG and incubated at 37°C until the density reached 2-4×10^7^ bacteria ml^−1^ before being harvested and resuspended in 10-15 ml of 20 mM phosphate buffer. *D. discoideum* wild-type AX2 and transformants were grown, harvested, resuspended in 1 ml of 20 mM phosphate buffer and transferred into a scintillation vial and incubated with shaking (150 rpm) at 21°C for 30 min to starve the cells. One millilitre of the harvested *E. coli* DSRed cells was added to the scintillation vial. At the time points T_0_ (0 min) and T_30_ (30 min), a 400 μl aliquot was removed and added to duplicate 10 ml Falcon tubes containing 3 ml of 5 mM NaN_3_. The cells in Falcon tubes were harvested, washed and lysed in 2 ml of 0.25% (v/v) Triton X-100 before the OD_640_ of the *E. coli* DsRed cells was measured using a spectrophotometer to allow calculation of the number of bacterial cells and the fluorescence per million bacteria (Maselli et al., 2002). The fluorescence for all transformants was measured in a Modulus fluorometer (Turner Biosystems, Sunnyvale, CA, USA) using a custom-manufactured module. Measurements were performed in duplicate at each time point, and the hourly consumption rate of bacteria by a single amoeba was calculated from the increase in fluorescence over 30 min, the fluorescence signal per million bacteria and the amoebal density.

##### Pinocytosis

The rate of pinocytosis was measured using the method developed by Klein and Satre (1986). *D. discoideum* cells were harvested, resuspended in HL-5 at the density of 1×10^7^ cells ml^−1^, and incubated at 21°C for 30 min, with shaking at 150 rpm. One hundred microlitres of fluorescein isothiocyanate (FITC)-dextran (20 mg ml^−1^) in HL-5 was added to 1 ml of *Dictyostelium* cell suspension. At the time points T_0_ (0 min) and T_70_ (70 min), a 200 μl aliquot was removed and added to duplicate 10 ml Falcon tubes containing 3 ml Sorensen buffer. The cells in Falcon tubes were harvested, washed and lysed in 2 ml of 0.25% (v/v) Triton X-100 before the fluorescence was measured in a Modulus Fluorometer using the ‘Green’ module. Measurements were performed in duplicate at each time point, and the hourly uptake of liquid medium by a single amoeba was calculated from the increase in fluorescence over 70 min, the fluorescence signal per 10 million bacteria and the amoebal density.

### Statistical analysis

Regression and correlation analysis was performed as previously described (Annesley et al., 2016).

## Supplementary Material

Supplementary information
