# Supplementary material for: The Parkinson's disease-associated protein DJ-1 plays a positive nonmitochondrial role in endocytosis in Dictyostelium cells
Source: Dis Model Mech. 2017 Oct 1;10(10):1261–71. doi: 10.1242/dmm.028084 (PMC5665451; doi:10.1242/dmm.028084)
Supplement: Supplementary information [file dmm-10-028084-s1.pdf]

Supplemental Information

Supplementary Figure S1.

a

VALUES OF COMPUTED PARAMETERS

Net charge of query sequence	:	-3
Analysed region	:	13
Number of basic residues in targeting sequence	:	3
Number of acidic residues in targeting sequence	:	0
**Cleavage site**	:	**not predictable**
Cleaved sequence	:	

HYDROPHOBIC SCALE USED

		GES	KD	GvH1	ECS
H17	:	0.835	1.359	0.027	0.529
MesoH	:	-0.973	0.319	-0.456	0.203
MμHδ_075	:	14.947	10.979	5.993	1.857
MμHδ_095	:	26.037	12.051	6.547	5.040
MμHδ_100	:	34.806	16.396	8.945	5.995
MμHδ_105	:	37.341	18.333	9.998	6.460
Hmax_075	:	6.650	15.633	2.117	4.422
Hmax_095	:	10.675	13.213	1.803	5.093
Hmax_100	:	19.100	19.700	3.370	6.460
Hmax_105	:	18.000	17.100	3.151	5.870

PROBABILITY of export to mitochondria: **0.1553**

b

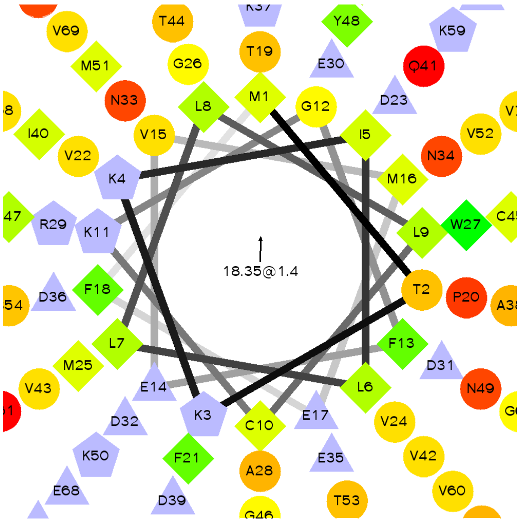

Supplementary Figure S1. Prediction of DJ-1's subcellular localization.

a: Prediction of DJ-1's subcellular localization using MitoProt II. The entire DJ-1 protein of 205 amino acids was used in this program and 13 residues from the N-terminus were analysed to predict probability of export to the mitochondria as mitochondrial targeting sequence is usually located in the N-terminus of proteins. A score of 0.1553 out of 1 was obtained, which suggests DJ-1 unlikely to be localized to the mitochondria.

The bottom panel of the table lists the scales used to calculate the hydrophobicity of the protein. H17: the 17-residue segment of highest hydrophobicity in the sequence. Increasing the value diminishes the probability of import; MesoH: the average of the maximal hydrophobicity of a protein over an extended sequence length; MμHδ: the maximal Eisenberg's hydrophobic moment with 6 angles of 75°, 95°, 100° and 105°, using a scanning window of 18 residues; Hmaxδ: the maximal hydrophobicity of each hydrophobic face in a helical structure. It is calculated for the 18 residues determined by the maximal MμHδ. To minimize the bias introduced by the scale, calculations have been made with up to four scales based in different amino acid residue properties. GES (Goldman, Engelman and Steitz scale): reflects the circumstances in which amino acid residues appear in proteins by quantifying quantifying the free energy of water/oil transfer for residues in an α-helical structure; GvH1 (Gunnar von Heijne scale 1): a statistical scale obtained from the amino acid residue frequencies in the central part of a transmembrane segment with respect to the nonmembranous stretches; KD (Kyte and Doolitle scale): considers values from water-to-vapour energy transfers and from internal/external distribution of amino acid residues; ECS (Eisenberg's Consensus scale): is a normalized average of the preceding 4 scales that mitigates the effects of outlying values in any one scale resulting from the peculiarities of particular methods (Claros & Vincens, 1996; http://ihg.gsf.de/ihg/mitoprot.html).

b: Prediction of DJ-1's subcellular localization using a Helical Wheel plot. The hydrophilic residues are shown as circles, hydrophobic residues as diamonds, potentially negatively charged residues as triangles, and potentially positively charged as pentagons. Hydrophobicity is color coded: the most hydrophobic residue is green, and the amount of green decreasing proportionally to the hydrophobicity, with zero hydrophobicity coded as yellow. Hydrophilic residues are coded red with pure red being the most hydrophilic (uncharged) residue, and the amount of red decreasing proportionally to the hydrophilicity. The potentially positively or negatively charged residues are light blue.

Supplementary Figure S2.

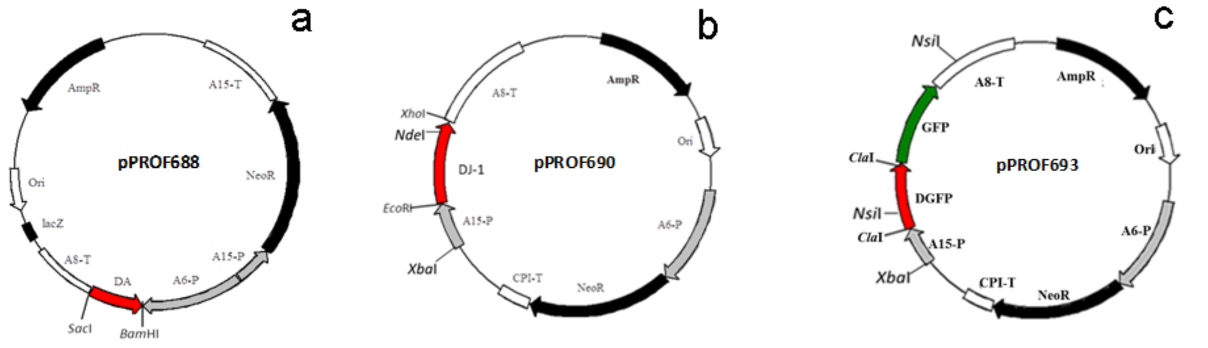

Supplementary Figure S2. Circular maps of DJ-1 constructs.

a: Circular map of construct pPROF688 with insertion of DJ-1 fragment (DA) into pDNeo2 A fragment of the DJ-1 gene (75-479 bp) was cloned into the *Sac*I *and Bam*HI restriction enzyme sites of pDNeo2 in the antisense orientation. Neo^R: G418 resistance which is controlled by the actin-15 promoter and terminator (A15-P and A15-T). AmpR: ampicillin resistance; lac Z operon: for blue/white screening. Ori: origin of DNA replication; The DA expression cloning cassette is regulated by the actin-6 promoter (A6-P) and the actin-8 terminator (A8-T).

b: Circular map of construct pPROF690 with replacement of Tet cassette with full DJ-1 in pPROF267 The Tet cassette in pPROF267 was replaced by the entire DJ-1 gene in the sense orientation using *Cla*I and *Xho*I restriction enzyme sites. G418 resistance cassette (Neo^R) in this construct is regulated by the A6-P promoter and the CPI-T terminator. The other gene cassettes designations are shown in Supplementary Figure 2a.

c: Circular map of construct pPROF693 with insertion of DJ-1 without the stop codon (DGFP) into pA15GFP The DGFP was cloned into the *Cla*I site of pA15GFP in the sense orientation. The DGFP:GFP gene is flanked by a functional promoter and terminator (A15-P and A8-T). The other gene cassettes designations are shown in Supplementary Figure 2b.

Supplementary Figure S3

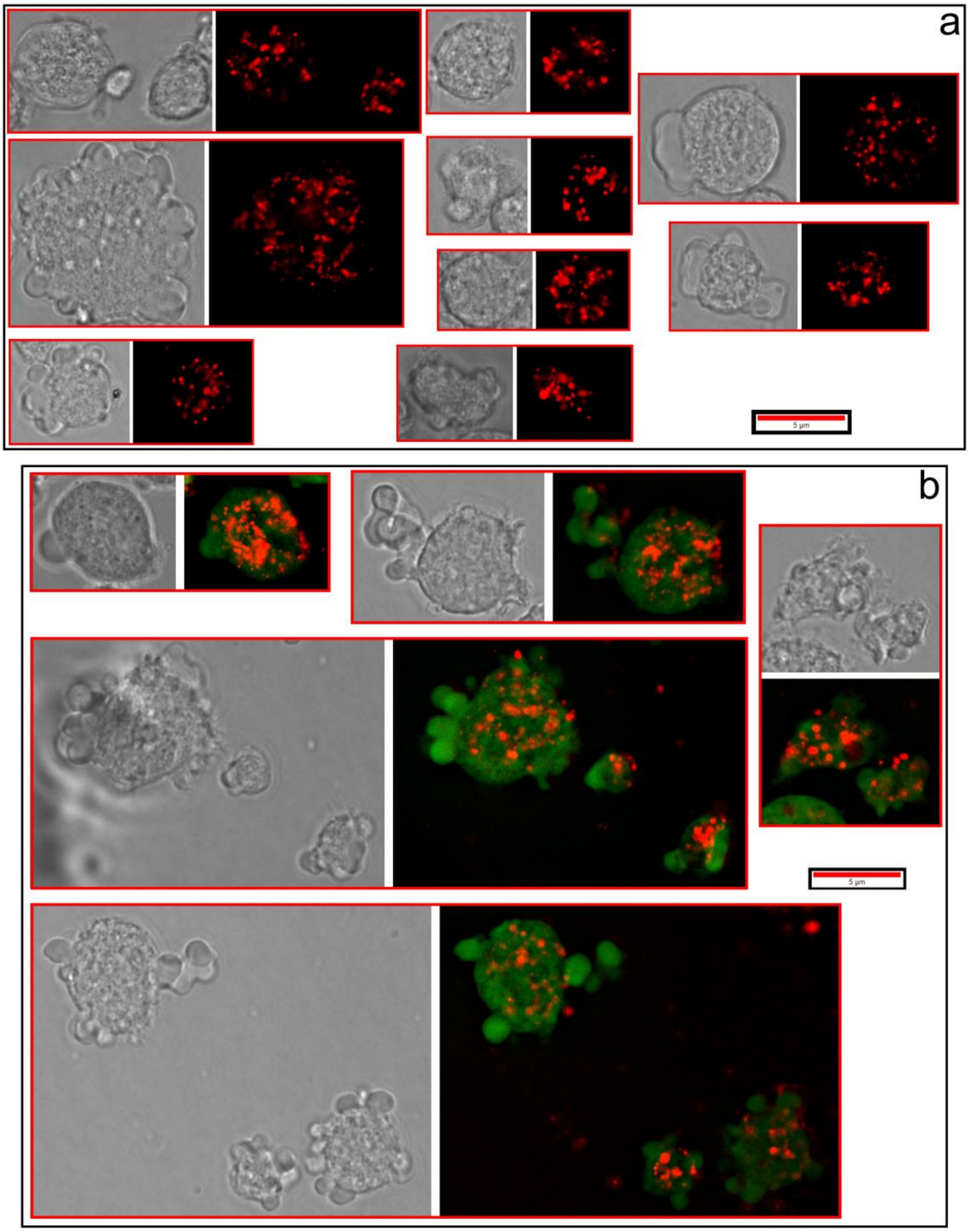

Supplementary Figure S3. The subcellular localization of DJ-1 in live *D. discoideum* cells**.** Phase contrast and deconvolution fluorescence images of live (a) parental and (b) DJ1:GFP expressing *D. discoideum* cells. Red fluorescence – Mitotracker Red staining of mitochondria. Green fluorescence - DJI:GFP; (DJ-1 fused to Green Fluorescent Protein expressed in strain HPF1246, stable transformant of parental strain AX2). Blue fluorescence – DAPI (4',6-diamidino-2-phenylindole) staining of nuclei was not performed as DAPI is not cell permeant. The DJ-1:GFP fusion protein was found throughout the cell in the cytoplasm with no enrichment in the mitochondria. Images were taken on an Olympus BX61 fluorescence microscope. Both wild type and transformed live cells in suspension showed significant levels of membrane blebbing under the conditions used. We do not know the reason for this but it did not affect the localization of DJ-1. After background subtraction and contrast adjusment, the image was deconvolved using the Olympus CellSens Dimension 1.16 software.

Disease Models & Mechanisms • Supplementary information

Supplementary Figure S4

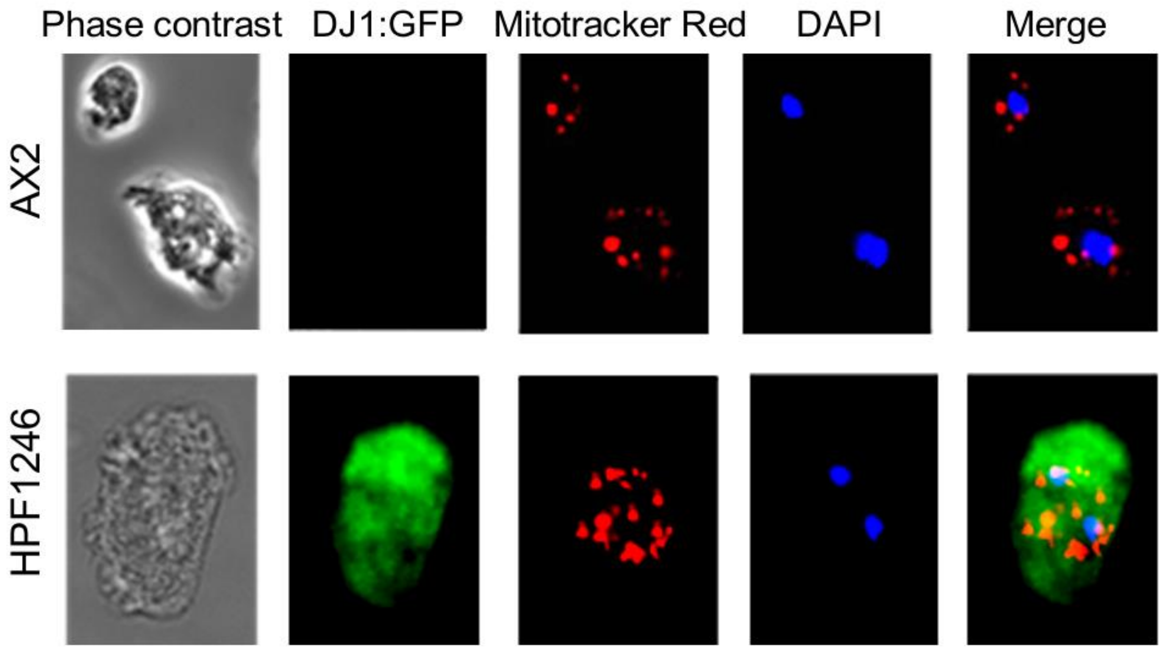

Supplementary Figure S4. Immunofluorescence microscopy of DJ-1:GFP in fixed, permeabilized *D. discoideum* cells. Phase contrast and epifluorescence images of parental (AX2) and DJ1:GFP (DJ-1 fused to Green Fluorescent Protein) expressing *D. discoideum* transformants (strain HPF1246). Cells were stained with Mitotracker Red, then fixed in 3.7% formaldehyde (in PBS) and permeabilized with ice-cold methanol, then immunostained and finally stained with DAPI (4',6-diamidino-2-phenylindole). PH: Phase contrast image of the *D. discoideum* cells; Mitotracker Red: Red fluorescence – Mitotracker Red staining of mitochondria. GFP: Green fluorescence - DJI:GFP detected by immunofluorescence using AlexFluor^{R}488-conjugated rabbit anti-GFP IgG antibody (Invitrogen^{TM}). DAPI: Blue fluorescence – DAPI staining of nuclei. Merge: the overlay of all the images. The DJ-1:GFP fusion protein was found throughout the cell in the cytoplasm with no enrichment in the mitochondria. Some larger Mitotracker Red-stained clusters of mitochondria are poorly resolved into individual mitochondria.

Supplementary Figure S5

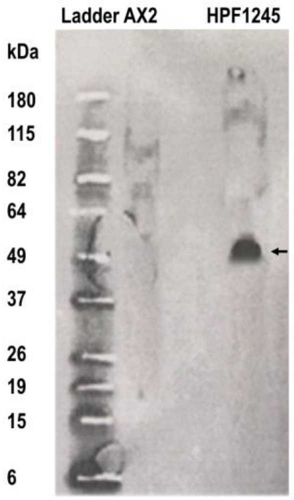

Supplementary Figure S5. Expression of GFP in a transformant expressing GFP-tagged DJ-1. Western blot of protein from AX2 (negative control) and HPF1245 expressing GFP-tagged DJ-1 (52 kDa - arrowed). Crude protein lysates were separated by SDS-PAGE on a 12% polyacrylamide gel and transferred to a Hybond^{TM}-P+ nylon (Amersham) membrane before washing, blocking and detection using Enhanced Chemifluorescence with anti-GFP-rabbit IgG fraction (Alexa Fluor 488 conjugate) diluted 1/500 in blocking buffer. No GFP could be detected in the parental AX2 strain.

Disease Models & Mechanisms • Supplementary information

Supplementary Figure S6

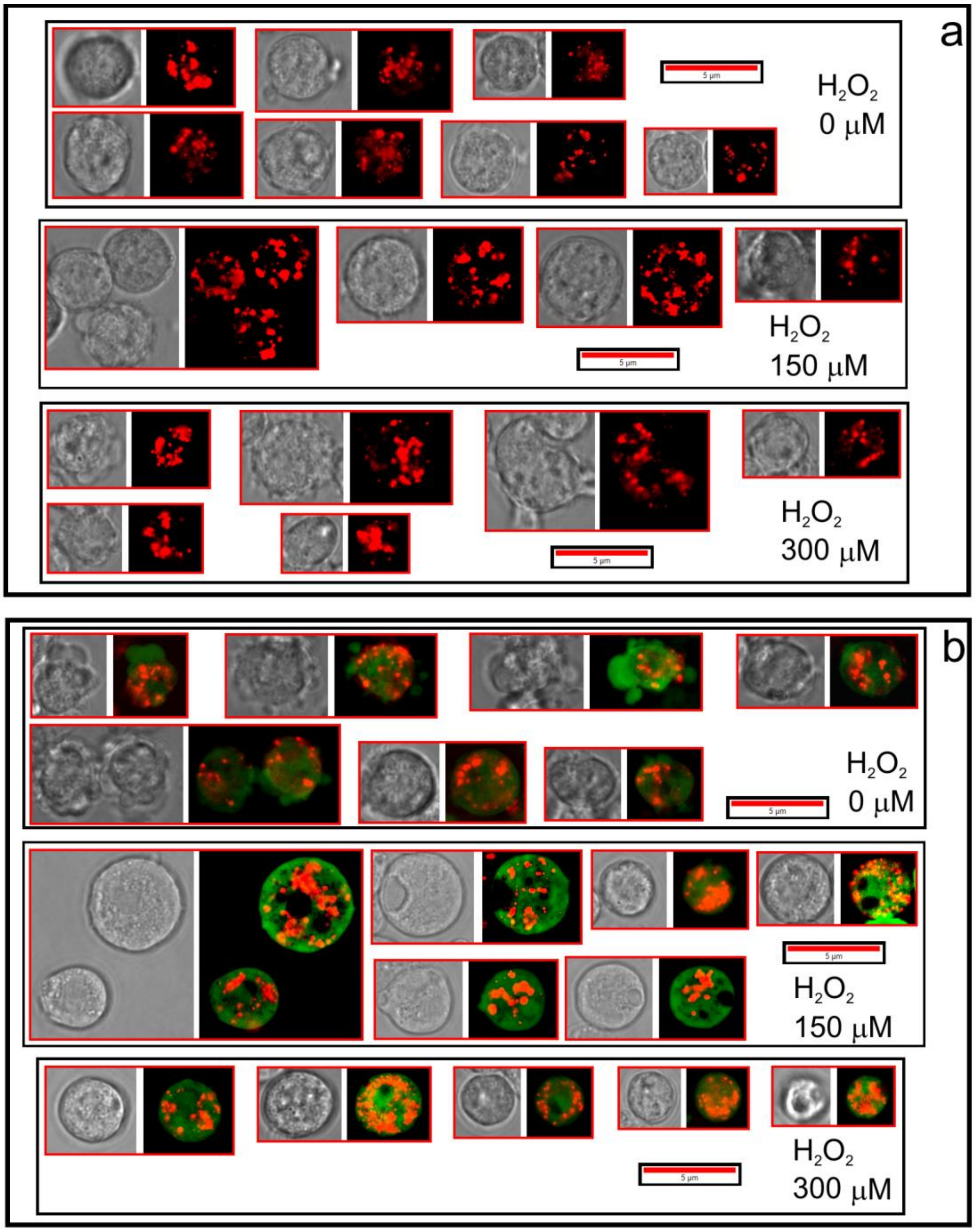

Supplementary Figure S6. The subcellular localization of DJ-1 in live *D. discoideum* cells is unaffected by oxidative stress. Phase contrast and deconvolution fluorescence images of live (a) parental and (b) DJ1:GFP expressing *D. discoideum* cells. Red fluorescence – Mitotracker Red staining of mitochondria. Green fluorescence - DJI:GFP; (DJ-1 fused to Green Fluorescent Protein expressed in strain HPF1246, stable transformant of parental strain AX2). The DJ-1:GFP fusion protein was found throughout the cell in the cytoplasm with no enrichment in the mitochondria even after 24 h exposure to H_2O_2 at concentrations that inhibit growth. Images were taken on an Olympus BX61 fluorescence microscope. After background subtraction and contrast adjustment, the image was deconvolved using the Olympus CellSens Dimension 1.16 software.

Supplementary Figure S7

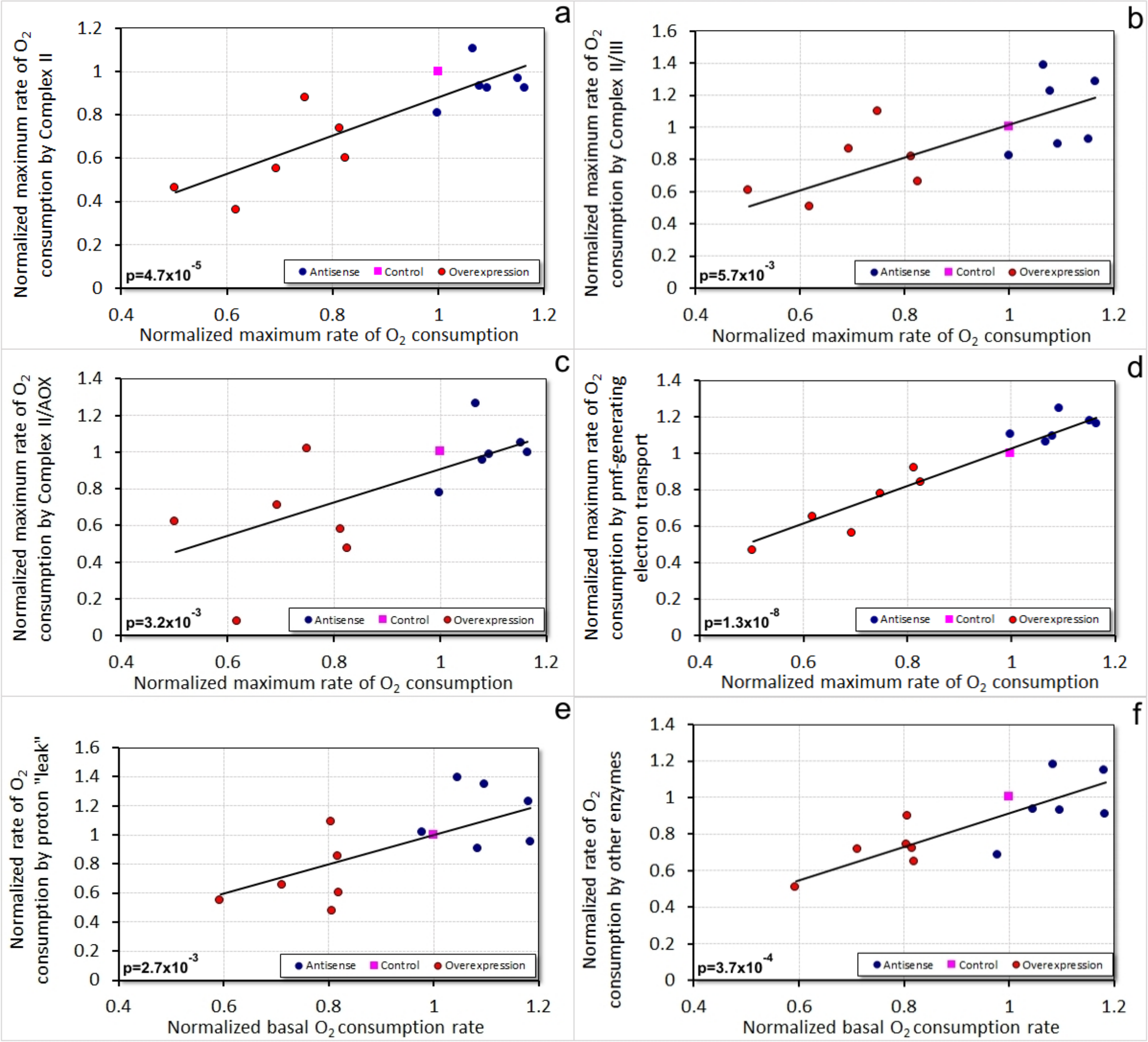

Supplementary Figure S7. The relationship between basal and maximal uncoupled respiration rates and key components thereof. The proportions of O_2 consumption contributed by individual components of basal and maximal CCCP-uncouped respiration are unaffected by DJ-1 knockdown or overexpression, so that the points in both groups of strains and the control strain all lie on the same regression line (line slopes not significantly different in multiple regression analysis). Key examples shown in the paper in Figure 8 are the portion of basal respiration used for ATP synthesis and the contribution to maximal CCCP-uncoupled respiration by Complex I.

Shown here are the relationships between maximal respiration and the individual contributions by **(a)** Complex II activity, comprised of **(b)** the component that is coupled to Complex III and **(c)** the component coupled to the *Dictyostelium* alternative oxidase (AOX). The proton-motive force generating portion of the maximum uncoupled respiration is the sum of Complex I and Complex II/III contributions **(d)** and provides energy for ATP synthesis and other mitochondrial processes such as protein, ion and metabolite transport. In intact cells the contributions to basal respiration include **(e)** the proton “leak” (“consumption” of mitochondrial respiratory energy by processes other than ATP synthesis eg. protein import) and **(f)** direct oxygen consumption by other cellular enzymes (oxidases and oxygenases).

If a given respiratory complex were functionally impaired in the DJ-1 antisense-inhibited or overexpression strains, it would contribute a different (smaller) fraction of respiratory O_2 consumption and so lie on a different regression line. Multiple regression analysis showed that this was not the case and that the relative contributions of individual respiratory complexes were unaffected by changes in the level of DJ-1 expression.

Each point represents the average from 2 to 17 independent experiments on a given strain, each experiment involving 4 technical replicates (separate wells in the assay plate). All regressions were highly significant (significance probabilities shown in each panel margin).

Disease Models & Mechanisms • Supplementary information

Supplementary Figure S8

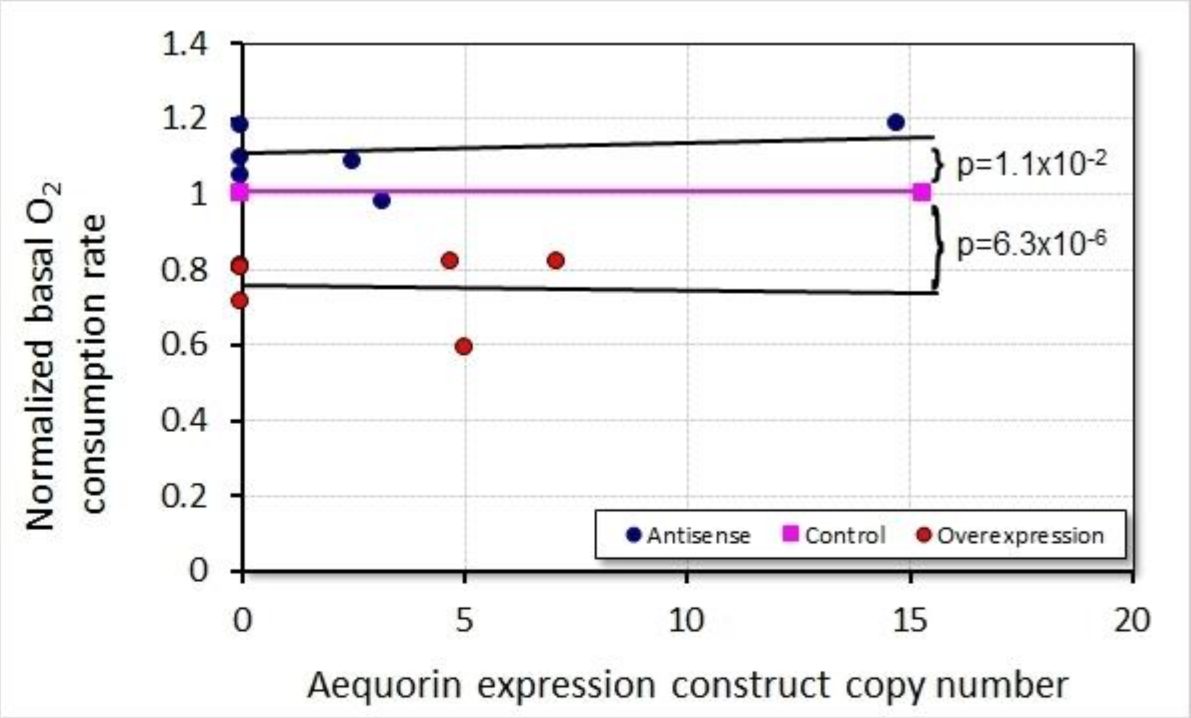

Supplementary Figure S8. The expression of aequorin has no effect on basal mitochondrial respiration, but DJ-1 knock down activates and DJ-1 overexpression inhibits respiration. Copy numbers of pPROF120, an apoaequorin expression construct, were determined by qPCR and the basal respiration rates determined and normalized against the average for the cognate control strains (AX2, 0 copies; HPF401, 153 copies). Multiple regression analysis showed that there was no significant correlation with copy number, but that the intercepts for the DJ-1 antisense and DJ-1 overexpression strains were respectively greater or less than 1 (ie above or below the controls, significance probabilities shown). In a separate multiple regression analysis testing the effects of the copy numbers of both the aequorin and DJ-1 constructs on basal respiration, the aequorin expression index (pPROF120 copy number) had no significant effect ($p>0.1$), but the effect of DJ-1 expression was copy number dependent and highly significant ($p=2.5 \times 10^{-4}$).

Supplementary Table 1.

Gene	Mitochondrial	ER	Elsewhere	Prediction
DJ-1	0.00	0.47	0.52	Possible ER

Prediction of DJ-1's subcellular localization using Predotar. The program Predotar provides a probability estimate as to whether the sequence of interest contains a targeting sequence for mitochondria, ER or other organelles. The number in the "elsewhere" column refers to the sum of the probabilities of DJ-1 being in organelles other than the mitochondria or ER, such as Golgi complex, nucleus *etc*. (Small *et al*., 2004; https://urgi.versailles.inra.fr/predotar/predotar.html).

References:

Claros, M.G. & Vincens, P. (1996) Computational method to predict mitochondrially imported proteins and their targeting sequences. *Eur J Biochem.* **241,** 779-86.

Small, I., Peeters, N., Legeai, F. & Lurin, C. (2004) Predotar: A tool for rapidly screening proteomes for N-terminal targeting sequences. *Proteomics* **4,** 1581-90.

Disease Models & Mechanisms • Supplementary information
